# A 7-Year History of Necrobiotic Xanthogranuloma following Asymptomatic Multiple Myeloma: A Case Report

**DOI:** 10.1155/2011/927852

**Published:** 2011-04-10

**Authors:** Siriluk Inthasotti, Rungsima Wanitphakdeedecha, Jane Manonukul

**Affiliations:** ^1^Department of Dermatology, Faculty of Medicine Siriraj Hospital, Mahidol University, Bangkok 10700, Thailand; ^2^Department of Pathology, Faculty of Medicine Siriraj Hospital, Mahidol University, Bangkok 10700, Thailand

## Abstract

Necrobiotic xanthogranuloma (NXG) is a rare destructive xanthomatous granuloma with chronic, indolent, and progressive course. The morbidity and mortality are the results from wound complications and associated disorders. Because of its strong association with monoclonal gammopathy and multiple myeloma, early recognition of disease is mandatory to monitor and prevent systemic involvements of hematologic malignancies.

## 1. Introduction

 Necrobiotic xanthogranuloma (NXG) is a rare nonLangerhans cell histiocytosis. Approximately 100 cases have been reported worldwide [1]. Clinically, it is characterized by multiple yellowish to reddish-brown nodules and plaques develop on periorbital regions, trunk, and extremities. Most lesions are asymptomatic but sometimes may ulcerate (40–50% of cases). Hematologic malignancy is the most important associated findings. Monoclonal gammopathies are found in 80–90% of cases [2–5]. 

On microscopic examination, it is characterized by the extensive area of necrobiosis surrounded with granulomatous infiltration from dermis to subcutis. Bizarre giant cells, Touton giant cells, foamy histiocytes, and cholesterol clefts are frequently found in necrobiotic area [6–8].

Necrobiotic xanthogranuloma may be difficult to diagnose because it is often asymptomatic and slowly progressive. If the patients present with only periorbital lesions, they may be misdiagnosed as xanthelasma and may not receive proper investigations and/or schedule followup for hematologic malignancies. In addition, surgical treatment in these patients may cause more complications.

Therefore, it is important to recognize the course of this rare disease in order to plan for long-term management and followup. Early detection of systemic involvements of either hematologic malignancies or necrobiotic xanthogranuloma itself is necessary.

## 2. Case Report

A 64-year-old woman presented with a 7-year history of multiple asymptomatic yellowish to reddish-brown nodules and plaques gradually occurred on both legs and arms. Few asymptomatic periorbital lesions had developed for the last few years. The first clinical impression was xanthelasma and granuloma annulare. Initial excisional biopsy was performed and diagnosed as subcutaneous granuloma annulare. Other investigations were done and showed hypercholesterolemia that was effectively controlled by lipidlowering agents. Some lesions were treated with intralesional and topical corticosteroids without any clinical improvement. The second biopsy was then performed and a diagnosis of xanthogranuloma was established. The lesions located on her both forearms, legs, and periorbital regions have progressively enlarged. Later on, the patient was referred to our hospital for dermatologic consultation.

**Figure 1:**
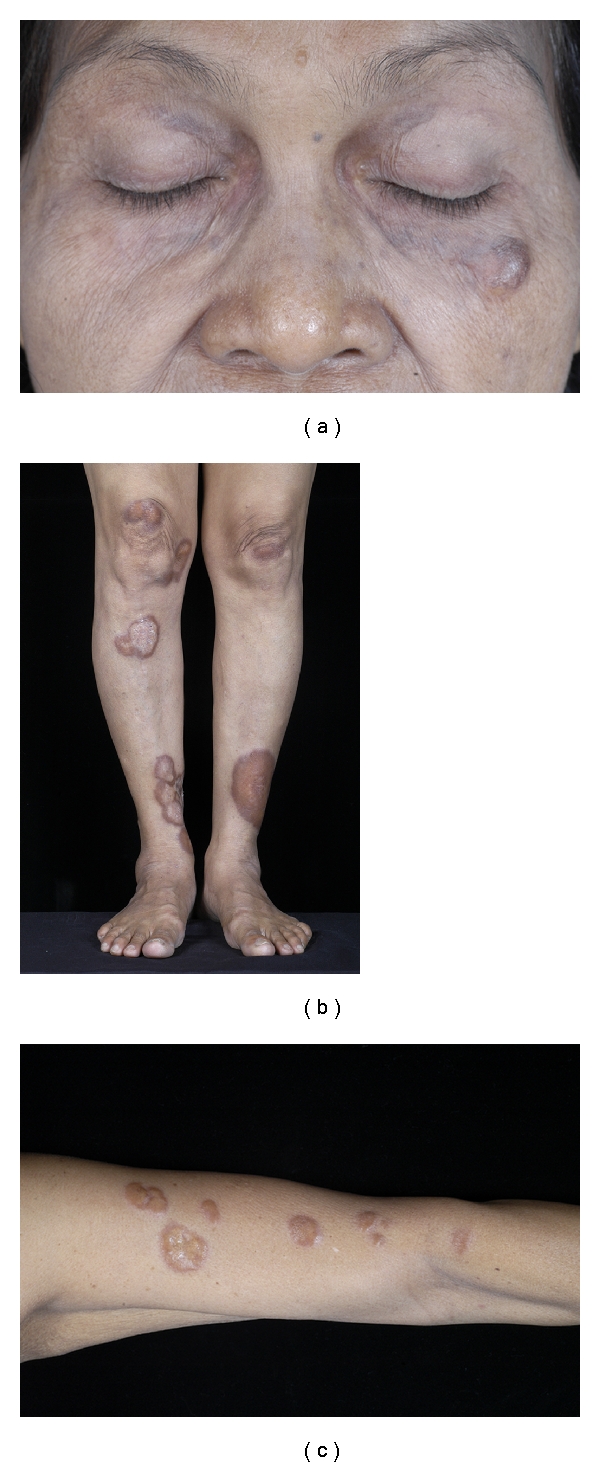
Physical examination demonstrated multiple yellowish to reddish-brown nodules and plaques on face (a) and extremities (b). Some lesions were observed to have central ulceration and atrophy (c).

Physical examination showed multiple yellowish to reddish-brown nodules and plaques on face and extremities (Figures 1(a) and 1(b)). Some lesions were observed to have central ulceration and atrophy distributed on her legs and forearms (Figure 1(c)), healing with scar. The provisional differential diagnoses included necrobiotic xanthogranuloma, atypical necrobiotic lipoidica, and diffuse plane xanthoma.

**Figure 2:**
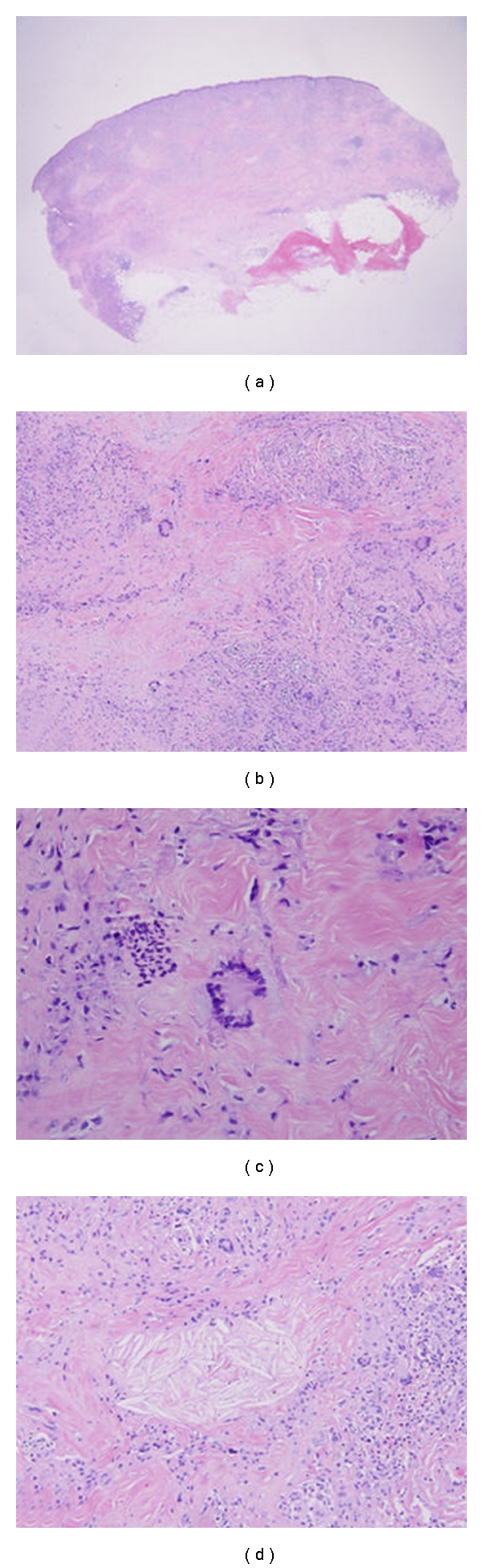
Histopathology (Hematoxylin & Eosin staining) revealed diffuse infiltration from papillary dermis to superficial subcutaneous fat by mononuclear cells (H&Ex12.5) (a). Palisaded granuloma with necrobiosis is also noted (H&Ex200) (b). Xanthoma cells and Touton giant cells (c) and cholesterol clefts (d) are seen (H&Ex400).

Skin biopsy was again performed and revealed palisaded granulomas containing foreign body type, Touton giant cells, foamy histiocytes, surrounding large necrobiotic areas that extended from the dermis to subcutis. Cholesteral clefts were found in the center of necrobiotic area (Figure 2). The findings previously described were favorable of necrobiotic xanthogranuloma (NXG). 

When NXG was diagnosed, other investigations for underlying hematologic malignancies were performed. Serum protein electrophoresis demonstrated monoclonal spike in gammaglobulin region, suggesting monoclonal gammopathy. Immunoelectrophoresis also showed IgG lambda monoclonal gammopathy. There was no osteolytic lesion in bone survey. Lastly, bone marrow study was compatible with multiple myeloma. 

In this patient, the final diagnosis was NXG associatied with asymptomatic multiple myeloma. Appropriate treatments for this condition included long-term closed followup for early detection of systemic involvements from multiple myeloma and necrobiotic xanthogranuloma itself. Intralesional corticosteroids have been employed to treat cutaneous lesions with mild clinical improvement.

## 3. Discussion

Necrobiotic xanthogranuloma (NXG) is a rare disorder, approximately 100 cases reported in the literature [1]. It is a chronic, progressive, multiorgan involved disease of unknown etiology [8]. Pathogenesis is still unclear. Age of onset is average at the sixth decade ranging from 17–85 years without sex predilection [3, 4]. NXG is clinically characterized by multiple yellowish to reddish brown nodules which slowly enlarge into plaques with yellowish hue and telangiectasia. 43% of cases developed central atrophy and ulceration.

The most common site of involvement is the face especially periorbital areas (85% of cases) [3, 4]. Most of the skin lesions initially present on the extremities or trunk [2]. The lesions are varying in size ranging from 0.5 to 20 cm. Oral mucosal lesions may be presented [9]. The periorbital lesions are the most characteristic sign in this condition starting with xanthelasma-like papules which progress into plaques. Most of cutaneous lesions are asymptomatic, but painful or burning sensation may be observed. 

Ophthalmologic manifestations can be seen in 50–80% of cases including conjunctival, corneal, and scleral involvements. Orbital masses and periorbital edema from xanthomatous inflammation of periorbital tissue have been reported [2, 10]. Magnetic resonance and axial computed tomography may be necessary in some patients to demonstrate ocular involvement.

NXG is now considered as a systemic disease. Internal organ involvements have been reported including spleen, heart, lung, kidney, intestine, ovary, larynx, pharynx, skeletal muscles, and the central nervous system. Lymphadenopathy is occasionally presented [3–5, 8, 11]. However, internal organ involvements in most cases are asymptomatic and diagnosis is established only in postmortem biopsy.

Hematologic and lymphoproliferative malignancies are the most important associated systemic disorders which develop approximately 2.4 years after the onset of skin lesions [1]. 80–90% of cases demonstrate monoclonal gammopathy (IgG Kappa for 60% and IgG Lambda for 26%); however, only 10% of cases will develop multiple myeloma [2, 12]. Other related conditions that can be found with NXG included Hodgkin's disease, non-Hodgkin's lymphoma, chronic lymphocytic leukemia, myelodysplastic syndrome, macroglobulinemia, cryoglobulinemia, and amyloidosis. Incisional biopsy is recommended in every suspicious patient to establish the diagnosis although the risk of wound complications is increased in NXG [8].

Histopathologic differential diagnoses include dissimilar diseases that can possess both necrobiotic areas and granulomas such as granuloma annulare (GA), necrobiotic lipoidica (NL), palisaded neutrophilic dermatosis (PND), and rheumatoid nodule (RN). Necrobiotic xanthogranuloma (NXG) differs from GA, NG, PND, and RN by the presence of prominent necrobiosis and often accumulating into large areas. Touton and foreign body type giant cells and cholesterol clefts are also obvious. In GA, the necrobiosis is usually focal and smaller and often contains less Touton giant cells and cholesterol clefts located in upper and mid-dermis. In NL, the necrobiotic areas are elongated, usually arranging in the step-ladder, horizontal orientation. PND and RN can be distinguished easily from NXG because of the presence of innumerable neutrophils in the infiltrate in the former and deposition of fibrin inside the necrobiotic areas in the latter.

Necrobiotic areas surrounded by granulomas in GA, PND, NL, and NXG can be found broadly from dermis to subcutis, while the necrobiotic areas in GA are always accumulated in focal area surrounded by lymphocytes and giant cells. In PND, neutrophils must be predominantly found. And multiple areas of palisaded granulomas distributing in horizontal orientation are characteristics of NL. Therefore, the diagnosis of GA, PND, and NL should be excluded. In NXG, the large necrobiotic area, Touton giant cells and cholesterol cleft can always be found, but rarely in NL. And this patient should be diagnosed as NXG. 

Diffuse plane xanthoma (DPX) was one of the clinical differential diagnoses in this patient. Histological examination of DPX should reveal large sheets and clusters of foamy cells, single and in small groups, diffusely scattered throughout the dermis, occasionally they may appear predominantly in a perivascular location [13]. Touton giant cells are rarely present in DPX. Histopathologic features observed in our case are not consistent with those in DPX. 

The clinical course of NXG is often chronic, progressive, and indolent. The prognosis is uncertain but generally good, depending on the severity of extracutaneous involvements, the presence of visceral malignancies, and wound complications. Patients should undergo for long-term periodic examination for hematologic and other associated malignancies [5, 9, 14]. Multiple myeloma developing in the patients with NXG seems to present with a relatively benign behavior. Ugurlu et al. found that 100% and 90% of patients with NXG and multiple myeloma could survive at least 10 and 15 years, respectively [2–4]. 

Currently, there are no randomizedcontrolled studies for therapeutic regimens. Chemotherapy is the most frequently used treatment and generally effective in modifying a disease course. Chlorambucil appears to be the most effective treatment for patients with extensive cutaneous lesions [15–17]. Other systemic agents have been employed with some improvement including systemic steroid [18, 19], chlorambucil plus systemic corticosteroids, [7] cyclophosphamide, [20] melphalan, [21, 22] melphalan plus systemic corticosteroids, [23–25] azathioprine plus systemic corticosteroids, [26] thalidomide [27], and interferon-*α*2b [7, 28]. All treatments can produce remission of paraproteinemia as well as skin lesions but, unfortunately, cannot prevent the evolution to multiple myeloma.

Elners and coworkers demonstrated that intralesional injection of triamcinolone acetonide was effective and safe treatment for orbital NXG in adults [29]. Surgical excision may be benefits for localized cutaneous lesions, except lesions on periorbital area, because of high rate of recurrence, stimulation of lesional activity, and scar formation resulting in eyelids retraction. Cryotherapy and radiotherapy have been tried without satisfied results. Thalidomide may be an interesting option for recalcitrant skin lesions [12, 27]. 

In our patient, monoclonal gammopathy has been detected after cutaneous manifestations for 7 years without symptomatic multiple myeloma or other systemic involvements. However, life-long, physical examination, and laboratory investigations for malignancy surveillance should be performed periodically.
